# The primary photoreaction of channelrhodopsin-1: wavelength dependent photoreactions induced by ground-state heterogeneity

**DOI:** 10.3389/fmolb.2015.00041

**Published:** 2015-07-22

**Authors:** Till Stensitzki, Vera Muders, Ramona Schlesinger, Joachim Heberle, Karsten Heyne

**Affiliations:** Institute of Experimental Physics, Free University BerlinBerlin, Germany

**Keywords:** *Ca*ChR1, retinal, isomerization, femtosecond pump-probe spectroscopy, reaction model, ground-state heterogeneity

## Abstract

The primary photodynamics of channelrhodopsin-1 from *Chlamydomonas augustae* (*Ca*ChR1) was investigated by VIS-pump supercontinuum probe experiments from femtoseconds to 100 picoseconds. In contrast to reported experiments on channelrhodopsin-2 from *Chlamydomonas reinhardtii* (*Cr*ChR2), we found a clear dependence of the photoreaction dynamics on varying the excitation wavelength. Upon excitation at 500 and at 550 nm we detected different bleaching bands, and spectrally distinct photoproduct absorptions in the first picoseconds. We assign the former to the ground-state heterogeneity of a mixture of 13-*cis* and all-*trans* retinal maximally absorbing around 480 and 540 nm, respectively. At 550 nm, all-*trans* retinal of the ground state is almost exclusively excited. Here, we found a fast all-*trans* to 13-*cis* isomerization process to a hot and spectrally broad P_1_ photoproduct with a time constant of (100 ± 50) fs, followed by photoproduct relaxation with time constants of (500 ± 100) fs and (5 ± 1) ps. The remaining fraction relaxes back to the parent ground state with time constants of (500 ± 100) fs and (5 ± 1) ps. Upon excitation at 500 nm a mixture of both chromophore conformations is excited, resulting in overlapping reaction dynamics with additional time constants of <300 fs, (1.8 ± 0.3) ps and (90 ± 25) ps. A new photoproduct Q is formed absorbing at around 600 nm. Strong coherent oscillatory signals were found pertaining up to several picoseconds. We determined low frequency modes around 200 cm^−1^, similar to those reported for bacteriorhodopsin.

## Introduction

Microbial rhodopsins comprise a large family of light-driven ion pumps and sensors. In 2002, a new functionality of microbial rhodopsins was introduced by the discovery of a light-gated ion channel (named channelrhodopsin) in the eyespot of the green algae *Chlamydomonas reinhardtii* (Nagel et al., 2002). A year later, a second channelrhodopsin (*Cr*ChR2) was characterized, Nagel et al. (2003) which paved the way for the new field of optogenetics where action potentials are elicited in neurons simply by remote illumination (Fenno et al., 2011).

Common to all rhodopsins, the polypeptide folds into the membrane in the form of a seven-helical bundle with the retinal chromophore covalently attached to a conserved lysine to form a protonated Schiff base (SB). Electron microscopy provided the first structural information on *Cr*ChR2 and resolved the arrangement of the seven transmembrane helices (Muller et al., 2011). X-ray crystallography provided a high-resolution three-dimensional structural model of C1C2, a chimera of channelrhodopsin derived from *Cr*ChR1 (helices A–E) and *Cr*ChR2 (helices F, G) (Kato et al., 2012). It was shown by PELDOR spectroscopy that helices B and F move to open the cation channel under illumination (Krause et al., 2013; Sattig et al., 2013). Electron microscopy of the open state confirmed these helical movements among others (Muller et al., 2015).

All native channelrhodopsins (ChRs) are cation channels which share sequence homology and similar functionalities but differ in spectral sensitivity, photocurrent, and desensitization. The visible absorption of retinal in ChR1 from *Chlamydomonas augustae* (*Ca*ChR1) is red-shifted by 50 nm as compared to the widely employed *Cr*ChR2 (Hou et al., 2012). This spectral feature renders *Ca*ChR1 advantageous in optogenetic applications where an increased penetration depth of the excitation light is required.

Illumination of ChRs induces a cyclic reaction (Ritter et al., 2008; Lorenz-Fonfria and Heberle, 2014). Up to now, the photocycle of *Ca*ChR1 has been recorded only at ns time resolution. Akin to the photoreaction of *Cr*ChR2 (Bamann et al., 2008; Ernst et al., 2008) an early red-shifted P^590^_1_ intermediate with absorption maximum at ~590 nm appears, which decays into the P^380^_2_ intermediate with absorption peak at 380 nm. The long lifetime of the P^380^_2_ state in *Ca*ChR1 is the most striking difference to *Cr*ChR2 (Sineshchekov et al., 2013) (our unpublished observations). As the lifetime of P^380^_2_ correlates with the lifetime of the passive channel current, the P^380^_2_ intermediate represents the conductive state of *Ca*ChR1. A P_3_ intermediate is not observed in *Ca*ChR1 but minor contributions from an O-like intermediate at 600 nm appear (Sineshchekov et al., 2013). Intramolecular proton transfer occurs in *Ca*ChR1 (Sineshchekov et al., 2013; Ogren et al., 2015a) but with distinct differences to *Cr*ChR2 (Lorenz-Fonfria et al., 2013; Ogren et al., 2015b).

UV/VIS pump-probe spectroscopy shows *Cr*ChR2 relaxation on the S_1_ potential energy surface by 150 fs, followed by a decay with a time constant of 400 fs into a hot ground state P_1_ and the parent ground state. The hot P_1_ relaxes with a time constant of 2.7 ps to a thermally equilibrated P_1_, the first intermediate state of the photocycle (Verhoefen et al., 2010). This intermediate state is characterized by retinal in a 13-*cis* conformation and accompanied by conformational changes in the protein backbone (Neumann-Verhoefen et al., 2013). A slow relaxation pathway of 200 ps was observed (Verhoefen et al., 2010).

The configuration of retinal in the ground state of *Ca*ChR1 was identified by retinal extraction followed by isomer separation with high-performance liquid chromatography (HPLC). Similar to *Cr*ChR2, the retinal isomer composition of the ground state result in a mixture of ~70:30 all-*trans* to 13-*cis* retinal (Nack et al., 2009; Muders et al., 2014). Resonance Raman experiments showed that the vibrational bands in the C = C stretching region derive from a mixture of retinals, which were assigned to mostly all-*trans* and partially 13-*cis* retinal. The band assignment in the C–C stretching region to contributions of 13-*cis* retinal was inexplicit, therefore also 100% all-*trans* retinal in the ground state was discussed (Ogren et al., 2014).

Here, we present the first femtosecond VIS pump supercontinuum probe spectroscopic experiments on *Ca*ChR1. Blue-shifted and red-shifted excitations with respect to the visible absorption maximum were applied to resolve the early photoreactions of 13-*cis* and all-*trans* retinal containing populations of *Ca*ChR1.

## Materials and methods

**Figure 1:**
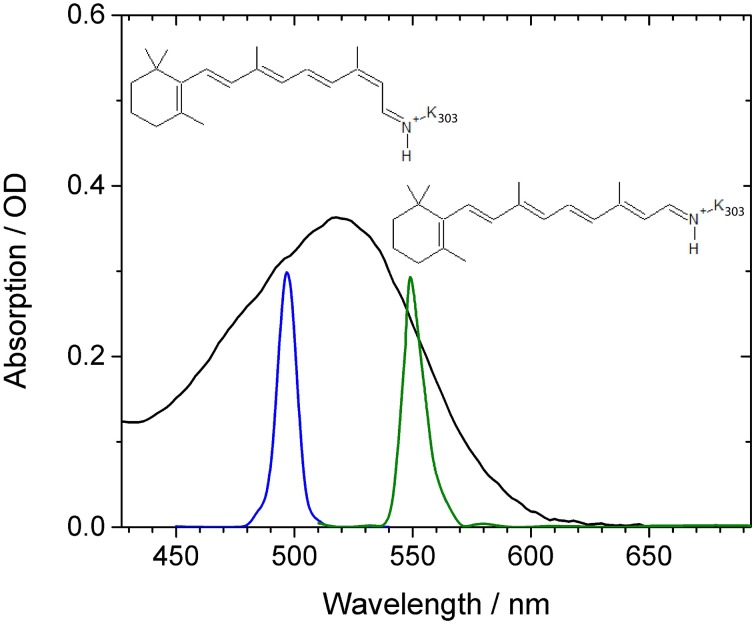
**Absorption spectrum of ***Ca***ChR1 (black line) as a function of wavelength**. Blue and green lines indicate the shape of the excitation pulses used in our experiments. The reported chromophore isomer structures of 13-*cis* retinal and all-*trans* retinal are inserted.

**Figure 2:**
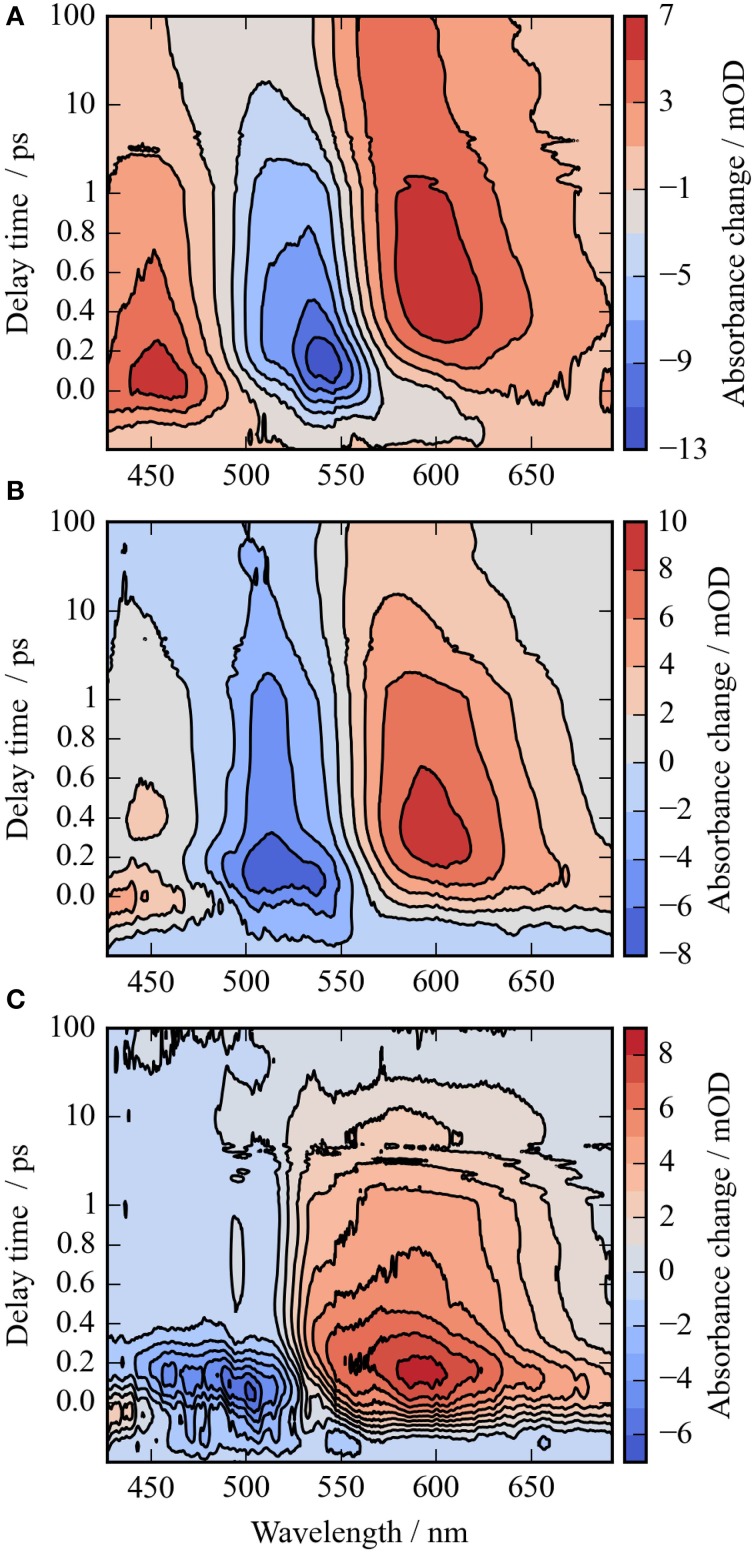
**Contour plots of the photoreaction dynamics of ***Ca***ChR1 upon excitation at 550 nm (A), 500 nm (B), and the difference of both datasets (C)**. Blue colors indicate negative signals, red colors positive signals. The contour plots display the absorbance difference in mOD upon excitation as a function of delay time and wavelength. The contour plots were smoothed in the time domain with Gaussian windows with a width of 4 points (FWHM of ~200 fs), the unfiltered dataset is depicted in Figure S2. The difference contour plot **(C)** is calculated by the direct difference of **(B)** and **(A)**.

*Ca*ChR1 was prepared as described (Lorenz-Fonfria et al., 2014; Muders et al., 2014). Briefly, the truncated *Ca*ChR1 gene (1–352 aa) was fused with a 10 × His-tag (GeneArt, Life Technologies) and was heterologously expressed in *Pichia pastoris* cells. The solubilized protein was purified on a Ni-NTA column (Macherey-Nagel, Germany) and concentrated to 46 mg/mL in buffer containing 20 mM Hepes, 100 mM NaCl, 0.05% DDM at pH 7.4. Two times 150 μL of the *Ca*ChR1 solution was placed between two CaF_2_ windows. The sample cell thickness was 100 μm, and the sample was rapidly moved perpendicular to the beam direction by a Lissajous scanner to provide a fresh sample at every shot. The spectral shape of the two selected femtosecond excitation pulses are plotted with the absorption spectrum of *Ca*ChR1 in Figure 1. Femtosecond laser pulses were generated starting from a fundamental femtosecond laser pulse delivered by a 1 kHz Ti:Sa laser system (Coherent Legend USP, 80 fs pulses at 800 nm). The fundamental beam was split into two parts for pump and probe pulse generation. The pump pulses were generated in a non-collinear optical parametric amplifier (NOPA). A sapphire white light supercontiuum was used as seed, amplified in a BBO crystal by frequency doubled pulses at 400 nm. We selected energies to excite the sample of about 0.4–0.5 μJ per pulse with a pump focus diameter of about 300 μm. At an optical density of 0.25 OD in the absorption maximum we excite about 10% of the sample. The fundamental for the probe pulses were first directed over an optical delay line, then focused into a 1 cm water cell generating the broadband white light supercontinuum from ~400 to ~1100 nm for probing. We selected probe wavelengths from 427 to 693 nm. We achieved fluctuations of below 1% standard deviation with a properly aligned water white light setup. Both beams were focused into the sample cell by a curved mirror. Behind the cell, the probe beam is collimated and passed through a short-pass filter (<750 nm) and a polarizer. The beam is than focused into a fiber connected with a grating spectrometer (Andor Shamrock 303i, 600 l/cm) equipped with a CCD camera system (2000 × 5 pixel, 0.35 nm/pixel, Stresing GmbH Berlin). The spectral resolution was below 0.5 nm. We used step sizes of 30 fs from −1 to 5 ps, and step sizes of log_10_ for longer delay times, and 8000 averages per data point. Every second pump beam was blocked by a chopper to record excited and not excited sample volumes alternatively. Since we found negligible polarization effects, we selected perpendicular polarization between pump and probe beam to reduce stray light. The time-zero was determined by recording the signal in pure CaF_2_ of the sample window. The delay with a maximal signal for each pixel was found and the resulting wavelength-delay curve was fitted with a 3rd order polynomial. For better visibility, the data shown in the contour plots in Figure 2 were smoothed in the time domain with Gaussian windows with a width of 4 points of 30 fs step size (corresponding to FWHM of 200 fs). This strongly reduces the oscillatory features. The unfiltered dataset is available in Figure S2. The instrument response function (IRF) determined to be 90 fs is governed by the pump pulse length (see Figure S6) (Kovalenko et al., 1999). The chirped water white light supercontinuum has negligible influence on the IRF after mathematical chirp correction Hence, the low frequency mode at 316 cm^−1^ with an oscillation period of 104 fs of our CaF2 windows could be well-resolved, and was used as an internal standard.

## Results

In Figure 1 the absorption spectrum of *Ca*ChR1 is presented with two spectrally different excitation pulse positions, and the two retinal chromophore conformations. The absorption spectrum peaks at 518 nm, exhibits a steeper decline at longer wavelengths compared to shorter wavelengths, and has a shoulder at around 470 nm. Raman studies showed that the retinal chromophore in ground-state *Ca*ChR1 adopts a mixture of 13-*cis* and all-*trans* conformations with a fraction of ~30 and ~70%, respectively (Muders et al., 2014).

Thus, different absorptions are expected for *Ca*ChR1 harboring 13-*cis* and all-*trans* retinal (Muders et al., 2014). The displayed absorption profile is broad and covers about 100 nm similarly as it was reported for dark-adapted bacteriorhodopsin and some bacteriorhodopsin mutants (Mowery et al., 1979; Harbison et al., 1984; Heyne et al., 2000). Assuming the same protein surrounding, and a simple particle in a box approach for the direction of the electronic transition dipole moment, the 13-*cis* retinal is expected to absorb at lower wavelengths due to a reduced length in one direction of its bent conjugated π-electron system of the ethylenic moiety compared to all-*trans* retinal (Aton et al., 1977; Fodor et al., 1989). Therefore, we expect complex photoreaction dynamics of *Ca*ChR1 with 13-*cis* retinal and with all-*trans* retinal upon excitation at 500 nm, while upon excitation at 550 nm at the low energy side of the absorption spectrum the photoreaction dynamics of *Ca*ChR1 containing all-*trans* retinal will dominate.

**Figure 3:**
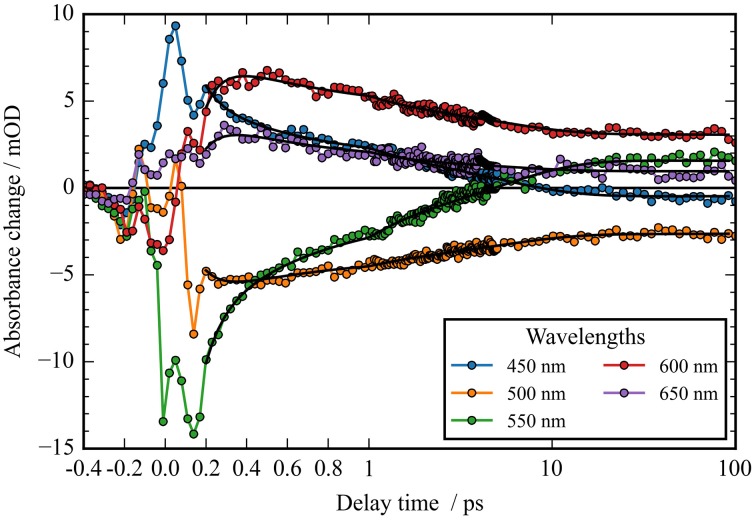
**Transients (dots) and simulated transients (black lines) upon excitation at 550 nm: the temporal change in absorption of ***Ca***ChR1 is plotted as a function of delay time after excitation**. Transient changes on the 100 femtosecond, sub picosecond, and picosecond time scales are directly visible.

**Figure 4:**
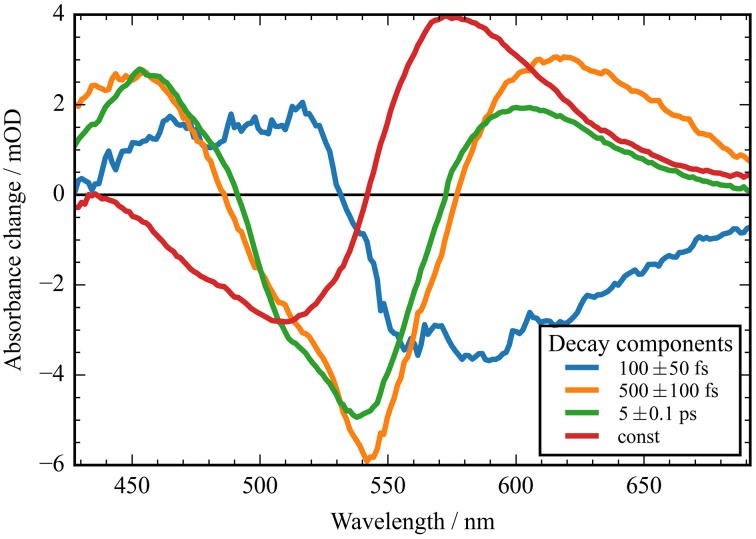
**Decay associated spectra (DAS) of the transient data upon excitation at 550 nm of ***Ca***ChR1**. Positive signals indicate decaying excited state and photoproduct absorption; negative signals indicate decaying bleaching absorption, rising photoproduct absorption, and stimulated emission decay. The fast component (blue line) exhibits no signature of the bleaching signal. The two components at 500 fs and 5 ps decay time exhibit similar spectral shapes with deviations at the high and low energy side. The red line displays the spectral difference between the bleaching signal and the photoproduct P_1_.

**Figure 5:**
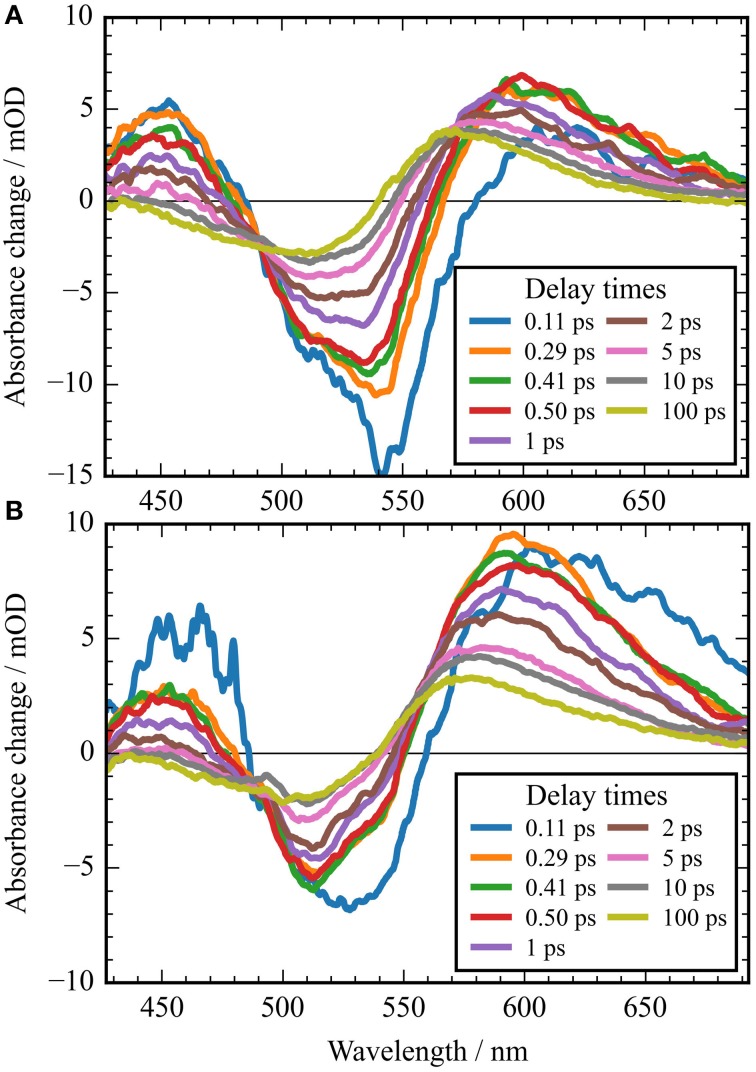
**Absorbance difference spectra of ***Ca***ChR1 upon excitation at 550 nm (A), and upon excitation at 500 nm (B)**. The first spectra at 0.11 ps could still be influenced by the instrument response function. Comparison of both spectra displays striking differences in the photoproduct region around 600 nm at picosecond delay times.

In Figure 2A the absorbance change of *Ca*ChR1 upon excitation at 550 nm is presented as a function of probe wavelength from 427 to 693 nm for different pump probe delay times. In this contour plot positive signals are found in the spectral region around 450 and 600 nm, while negative signals are visible around 540 nm. Upon excitation the initial absorption increase of excited state absorption (ESA) is found around 450 nm. The transient at 450 nm is plotted in Figure 3 (blue dots). The major part of the ESA signal exhibits a fast decay with time constant τ_1_ of 100 fs accompanied with a spectral narrowing displayed in Figure 2A, in conflict with a blue shift of the ESA. This points to relaxation or isomerization on the electronic excited state potential energy surface. The negative signal in Figure 2A exhibits a blue shift and decays at longer wavelengths on the same time scale. The major fraction of the transient at 550 nm (Figure 3, green dots) decays with τ_1_, a smaller fraction exhibits time constants of τ_2_ = (0.5 ± 0.1) and τ_3_ = (5 ± 1) ps. Decay associated spectra (DAS) representing decaying spectral features with a given time constant are displayed in Figure 4. The DAS of the time constant τ_1_ = 100 fs exhibits a positive signal from 427 to 490 nm, and a negative signal for longer wavelengths. At short wavelengths the positive signal shows the instantaneous ESA, while the negative signal represents stimulated emission decay and the rise of the first photoproduct. Note, there is no contribution matching the bleaching signal, indicating no back reaction to the parent ground state on this ultrafast time scale. We assign the time constant τ_1_ = (100 ± 50) fs to excited state decay accompanied by stimulated emission decay, and 13-*cis* photoproduct formation. The DAS for time constants of 500 fs and 5 ps exhibit very similar spectral features with a stronger signal for long and short wavelengths of the 500 fs component. This could be interpreted by involvement of the same electronic ground state showing broader spectral features of a hotter ground state at early delay times. A hot ground state is characterized by population of excited vibrations not relaxed to their thermal equilibrium. These populated non-thermal vibrations relax via intra- and intermolecular vibrational redistribution pathways on a picosecond time scale (Heyne et al., 2004a,b; Rey et al., 2004; Kozich et al., 2006; Shigeto et al., 2008). The positive signal contributions around 450 nm occur instantaneously upon excitation and persist up to 100 ps, as visible by the vanishing negative signal at 430 nm at 100 ps delay time (Figures 4, 5A). Since the bleaching signal has negative contribution at this spectral position, a positive band is also contributing there. Thus, the first thermally relaxed photoproduct P_1_ exhibits a broad spectral absorption from 427 to 693 nm with a maximum at about 560 nm (see Figure S1).

The rise of the vibrational excited (hot) photoproduct P_1_ within 100 femtoseconds is visible at 600 nm, and at 650 nm in Figures 2A, 3. After formation of the hot photoproduct P_1_ a relaxation occurs on the low energy and high energy side of the absorption on a time scale of τ_2_ = (500 ± 100) fs to a more cooled, but still hot photoproduct, which relaxes further with a time constant of τ_2_ = (5 ± 1) ps to the thermally relaxed P_1_. As a result of the cooling processes the positive absorption shifts to smaller wavelengths and the spectral feature narrows as displayed in Figure 2A. No stimulated emission signals were observed after 200 fs, corroborating the excited state decay with <100 fs.

The spectral integrated transient upon excitation at 550 nm is depicted in **Figure 7**. The spectral integrated transient provides information on the overall change in extinction coefficient. This assumption holds for integrated spectral ranges covering the whole contributing absorption band. This is fulfilled to a high extend in our measurements. Upon excitation at 550 nm a strong coherent contribution of the CaF_2_ sample cell window is clearly visible in **Figure 7** around time zero. We observe coherent oscillations in the first picoseconds with a period of ~100 fs. As shown in **Figure 7** the overall integrated transient rises within 100 femtoseconds to positive values, and stays nearly constant for picoseconds. Spectral integrated transients are not affected by spectral shifting, but are sensitive to new emerging species with different extinction coefficients. We see no significant signal change after 300 fs. Therefore, we assign the time constants of 500 fs and 5 ps to cooling processes of the photoproduct. As presented in Figure 4, the stimulated emission decays with a time constant of 100 fs. Since, the only detectable transition from the electronic excited state to another state is connected with the time constant of 100 fs, we assign this process to the all-*trans* to 13-*cis* isomerization.

It was reported that the initial photoreaction is independent of the excitation wavelength in *Cr*ChR2 (Verhoefen et al., 2010). For *Ca*ChR1, we see significant changes upon changing the excitation wavelength from the low energy side of the absorption band at 550 nm to the high energy side of the absorption band at 500 nm. The differences are best visible by comparing Figures 5A,B, as well as by comparing Figures 2A,B.

In Figure 2B the contour plot of the photoreaction dynamics upon 500 nm excitation is plotted. Again, there are instantaneous positive signals around 450 nm, instantaneous negative signals around 510 nm, and positive signals around 600 nm showing a delayed emergence. The negative signal exhibits features of a fast decaying fraction around 560 nm, pointing to a very small stimulated emission as compared to excitation at 550 nm. In addition, the bleaching signal peaks clearly at 510 nm continuing in position. The positive signal around 600 nm is much stronger compared to excitation at 550 nm. Since we expect to excite *Ca*ChR1 with 13-*cis* retinal as well as *Ca*ChR1 with all-*trans* retinal upon excitation at 500 nm, the photoreaction dynamics should consist of two parts. One part describes the photoreaction of *Ca*ChR1 with all-*trans* retinal, the other part the photoreaction of *Ca*ChR1 containing 13-*cis* retinal. Since the bleaching signals at 100 ps, where the primary photoreaction is nearly finished, shows identical spectral shape from 430 to 510 nm for excitation at 550 and 500 nm, we have a handle to compare both photoreactions directly. Therefore, the dataset excited at 500 nm was scaled by 1.4 to match the bleaching signals of both datasets at 100 ps delay time. Then, we subtracted the dataset upon excitation at 500 nm from the dataset upon excitation at 550 nm. The resulting difference is plotted as a contour plot in Figure 2C. The difference dataset has negative signals below ~520 nm with a maximum around 480 nm, and positive signals above 520 nm. Within the first 100 femtoseconds (τ_1_ < 300 fs) the negative signal exhibits a strong decay, while the positive signal decays with a blue shift on this time scale (Figure 2C).

The remaining positive signal decays with time constants of τ_2_ = (1.8 ± 0.3) and τ_3_ = (90 ± 25) ps to zero. The corresponding decay associated spectra (DAS) and transients are presented in Figure S7. The positive signal corresponding to τ_2_ exhibits a maximum at 590 nm and a broad absorption from 520 nm to wavelengths longer than 690 nm. A small negative contribution is found around 500 nm. The DAS corresponding to τ_3_ (DAS_3_) has a smaller amplitude with a maximum at 570 nm and positive signals from 490 to 690 nm. Small negative contributions are found around 450 nm. The spectral integrated signal in Figure S4 exhibits an instantaneous positive feature masked by oscillations, decaying with time constants <300 fs, 1.8 ps, and ~90 ps. Since spectral integrated signals are insensitive to spectral shifts, three time constants indicate three transitions of electronic states. Thus, we assign the significant DAS_2_ signal not to a cooling effect, but to a change of the electronic state properties.

The back reaction of the excited *Ca*ChR1 with 13-*cis* retinal to the parent ground state is nearly complete within 100 ps. This explains the nearly identical negative shapes of the absorption difference signals upon excitation at 500 and 550 nm.

At 100 ps delay time the spectral shape of the negative bleaching band signals are nearly identical for excitation at 500 and 550 nm (see Figure S5). Upon scaling of the bleaching bands for both excitations the photoproduct bands are rather similar, with band integrals differing by about 1.4, and absorption at longer wavelengths upon excitation at 500 nm. The intensity and spectral differences point to the existence of different photoproducts depending on the excitation energy. Increasing the excitation energy by shorter wavelengths introduce a higher amount of excess energy into the *Ca*ChR1 protein allowing for formation of photoproducts with higher ground-state energy and consequently red-shifted absorption. Ground-state heterogeneity of chromophore structures were reported for several photoreceptors (Gervasio et al., 1998; Sineshchekov, 2004; von Stetten et al., 2008; Mailliet et al., 2011; Kim et al., 2012; Ritter et al., 2013).

Examination of the photoreaction quantum yield is difficult and can only be roughly estimated by our data.

We estimated a quantum yield for the *Ca*ChR1 with all-*trans* retinal upon excitation at 550 nm to be higher than 0.25 and lower than 0.7. Excitation of the *Ca*ChR1 at 500 nm is lower compared to excitation at 550 nm.

**Figure 6:**
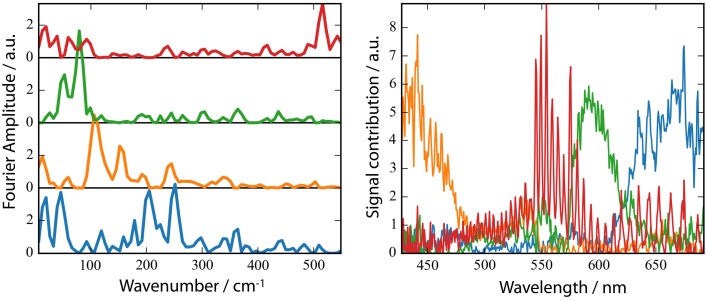
**Fast Fourier Transform (FFT) amplitudes of the residuals (A) and the spectral distribution of the components (B). (B)** non-negative matrix factorization (NMF) of the 2D matrix presented in Figure S3 of the FFT amplitudes mapped to 4 components. The number of components was inferred from the singular values of the matrix.

A striking property of the *Ca*ChR1 dynamics are the coherent oscillations visible in the transients (Figure 3), as well as in the contour plots (Figure S2). We obtained coherent oscillations up to 3 ps delay time by subtracting the simulated exponential dynamics from the dataset. The remaining residues were Fourier transformed and the amplitudes were plotted in Figure 6A. Figure 6B presents the spectral distribution of the Fourier components. Upon excitation at 550 nm we were able to identify oscillatory signals at about 80, 100, 150, 200, and 225 cm^−1^. The vibrations around 100, 150, 200, and 225 cm^−1^ occur at spectral positions connected to the electronic excited state, and also to the photoproduct absorption at long wavelengths. This could be interpreted in a way that these four vibrations constitute a part of the reaction coordinate in *Ca*ChR1, transferring the electronic excited state population to the first hot photoproduct P_1_. These vibrations at 100, 155, 200, and 225 cm^−1^ were assigned for all-*trans* retinal in solution to a ring torsion vibration, a chain methyl and ring torsion vibration, a methyl ring torsion vibration, and a chain bending and methyl ring torsion vibration, respectively (Prokhorenko et al., 2006). Two similar coherent vibrations at 195 and 226 cm^−1^ were reported to be crucial to optimize the photoisomerization reaction, while the coherent vibration of 155 cm^−1^ were reported to reduce the photoreaction quantum yield in bacteriorhodopsin (Polli et al., 2010; Johnson et al., 2014).

## Discussion

**Figure 7:**
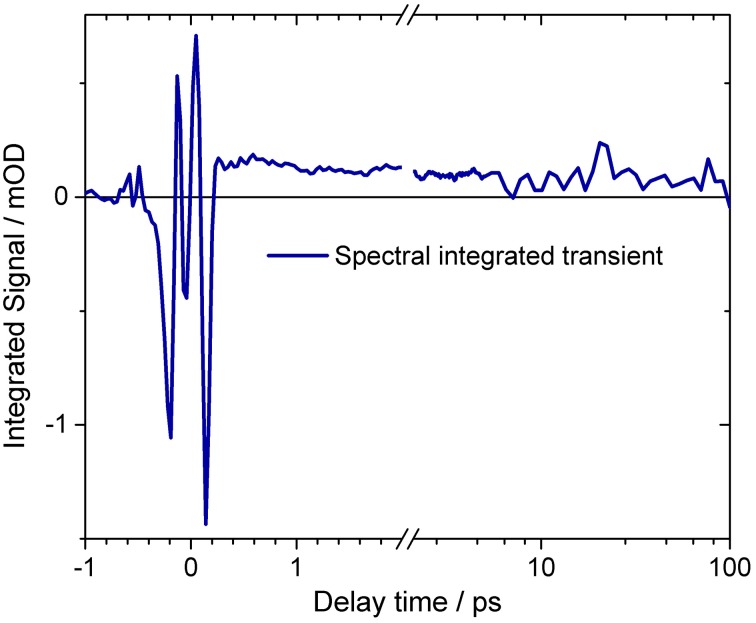
**Spectral integrated transient of the complete dataset upon excitation at 550 nm**. At delay times around time zero and before 200 fs strong oscillatory signals are visible. The mean signal directly after excitation is negative, rising to about 500 fs. On a picosecond time scale the signal stays nearly constant. The transient is plotted on a logarithmic scale for long delay times. A small signal decrease is observed. Small oscillatory signals are also visible for delay times after 200 fs.

**Figure 8:**
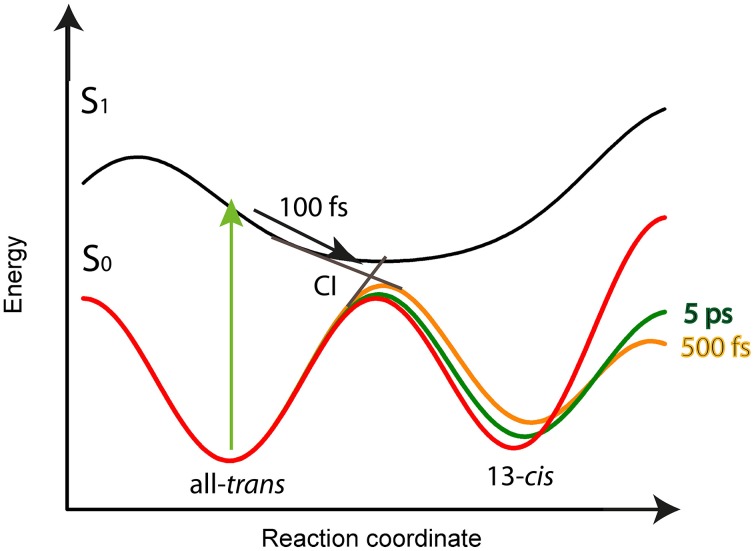
**Schematic potential energy surface as a function of the reaction coordinate for the ***Ca***ChR1 with all-***trans*** retinal**. After excitation (green arrow) the molecules relax within 100 fs on the S_1_ potential energy surface (black arrow) to the conical intersection (CI), indicated as straight lines from S_1_ to S_0_. Transition from S_1_ to S_0_ is accompanied with all-***trans*** to 13-***cis*** retinal photoisomerization. The excess energy excites vibrations of the chromophore and protein resulting in a softer ground-state potential energy surface (solid orange line) for the photoproduct P_1_ with 13-***cis*** retinal. Upon vibrational energy relaxation the ground-state potential energy surface becomes stiffer and more harmonic, thereby stabilizing the photoproduct (green line) until the fast relaxation process equilibrates (red curve). This process is connected with the same time constants as for parent ground-state recovery of 500 fs, and 5 ps.

Our model for the photoreaction of *Ca*ChR1 with all-*trans* retinal is presented in Figure 8:

Upon photoexcitation the all-*trans* retinal relaxes on the electronic excited state surface followed by an excited state decay and isomerization with a time constant of τ_1_ = (100 ± 50) fs via a conical intersection to the very hot photoproduct P_1_. Our decay associated spectrum (DAS_1_) in Figure 4 with a time constant of τ_1_ = (100 ± 50) fs is the only signature explaining the rise of the delayed positive signal around 600 nm. The excess energy is located in retinal and protein vibrations strongly coupled to the reaction coordinate. We propose a reaction coordinate consisting of several vibrations including the low-frequency vibrations at 205 and 225 cm^−1^. From the vibrational excited electronic hot P_1_ the *Ca*ChR1 with 13-*cis* retinal relaxes via cascaded energy redistribution processes (Heyne et al., 2004a) to the relaxed photoproduct P_1_ on a time scale of 500 fs and 5 ps. The initial P_1_ photoproduct absorption band exhibits a very broad absorption ranging from 427 to 690 nm. A significant narrowing of the spectrum especially at the low-energy side of the photoproduct absorption is observed on a time scale of 500 fs, followed by a narrowing of the spectrum at the high-energy side of the photoproduct absorption on a time scale of 5 ps. These spectral shifts without a change of extinction coefficient are visible in the spectrally resolved data (Figure 4), but not in the spectral integrated transient (Figure 7). These processes are indicated by the change of the potential energy surface shapes of P_1_ in Figure 8. Narrowing of P_1_ absorption upon vibrational relaxation is accompanied with the narrowing of the photoproduct potential well. Despite the fact, that we cannot directly detect the photoisomerization process as by time-resolved infrared spectroscopy, we were able to identify the excited state decay with a time constant of 100 fs and the emerged absorption of the photoproduct within 200 fs (Figure 2A). Thus, we conclude that the photoisomerization process is an ultrafast process with a time constant of (100 ± 50) fs. The strong red-shift of P_1_ absorption with 13-*cis* retinal can be explained by protein surrounding that is not equilibrated. The structural change of the chromophore, change of electric fields around the chromophore, and redistribution of the excess energy into the protein surrounding promotes the energy of *Ca*ChR1 ground state, resulting in a red-shifted absorption. These changes lead to an energetically elevated photoproduct ground-state P_1_ initiating the photocycle.

This early photoreaction mechanism is nearly identical to the photoreaction of rhodopsin (Schapiro and Ruhman, 2014). The chromophore relaxes from the Franck-Condon region within 100 fs and reaches the conical intersection. Within this time scale the stimulated emission vanishes completely in rhodopsin, as well as in *Ca*ChR1. In contrast, we see no ultrafast red-shift of the stimulated emission in *Ca*ChR1, probably due to a smaller extinction coefficient in *Ca*ChR1. With the disappearance of the stimulated emission the photoproduct absorption appears and shifts to higher energies in rhodopsin and in *Ca*ChR1.

The thermally equilibrated photoproduct P_1_ in *Ca*ChR1 absorbs maximally around 560 nm (Figure S1). The quantum yield of the forward reaction is roughly estimated to be between 0.25 and 0.7, as expected for retinal proteins. The photoproduct P_1_ is the first activated protein state of the photocycle of *Ca*ChR1.

The photoreaction is strongly influenced by coherent oscillations. Due to limited time resolution with an IRF of at least 90 fs we are able to identify strong coherent signals up to ~320 cm^−1^. We found oscillations resulted from low-frequency vibrations coupled to the photodynamics of *Ca*ChR1 with all-*trans* retinal at 205, 225, and 320 cm^−1^. We assign the vibration around 320 cm^−1^ to Raman vibrations of the CaF_2_ windows. The other low-frequency modes could be assigned to all-*trans* retinal vibrations (Prokhorenko et al., 2006) but further experiments with higher time resolution have to be performed to allow a precise assignment of frequency and phase of the involved vibrational oscillations.

In bacteriorhodopsin constructive and destructive interference effects of coherent vibrations drives the ultrafast isomerization process (Polli et al., 2010; Johnson et al., 2014). We expect a similar photoreaction mechanism for *Ca*ChR1 with all-*trans* retinal. Whether the quantum yield of the *Ca*ChR1 photoreaction can be optimized by coherent pulse shaping, has to be investigated in the future. As a result of the very fast isomerization process and formation of the hot electronic ground-state photoproduct, the chromophore and protein surrounding cannot follow this fast reaction speed. Thus, intra- and intermolecular energy redistribution processes on the sub picosecond and picosecond time scale transforms the system to the thermally equilibrated first photoproduct P_1_. Whether the different time scales can be assigned to intramolecular redistribution within the 13-*cis* chromophore and intermolecular redistribution between chromophore and protein has to be investigated by time-resolved infrared spectroscopy.

Comparison with reported photoreaction of *Cr*ChR2 reveals several similarities and differences. The photoproduct absorbs red-shifted to the bleaching band in *Ca*ChR1, and in *Cr*ChR2. In *Cr*ChR2 the excited state relaxes with 150 fs similar to the fast time constant in *Ca*ChR1 of 100 fs, but decays with a longer time constant of 400 fs to the photoproduct. The photoproduct cooling was determined to 2.7 ps, in contrast to *Ca*ChR1, where we observed two cooling processes with 500 fs and 5 ps. As visible in Figure 2A the spectral shifting in *Ca*ChR1 has not stopped completely at long delay times. In *Cr*ChR2 a similar spectral shift with a time constant of 200 ps was assigned to protein relaxation. For both systems the primary photoreaction process is assigned to all-*trans* to 13-*cis* retinal isomerization. Due to the blue-shifted absorption of *Cr*ChR2 with absorption maximum around 450 nm no positive signals from the ESA could be observed on the high energy side of the bleaching signal. In *Ca*ChR1, we detected the ESA on the high energy side of the bleaching signal, allowing for a direct separation of electronic excited state and photoproduct absorption. In contrast to *Cr*ChR2 we found strong oscillatory signals in *Ca*ChR1 and a dependence of the photoreaction dynamics on the excitation wavelength (Verhoefen et al., 2010).

Upon excitation at 550 nm we observe the expected photoisomerization of *Ca*ChR1 with all-*trans* retinal to the first photoproduct P_1_ cooling down on the picosecond time scale. Changing the excitation energy to 500 nm, results in significantly different photoreaction dynamics. The initial bleaching band is blue-shifted with respect to excitation at 550 nm, demonstrating heterogeneity of the *Ca*ChR1 ground state. Whether the origin of heterogeneity is due to differences in the chromophore structure or differences in the protein strongly interacting with the chromophore can be assessed by electronic or vibrational dynamics. The dynamics of the electronic states show distinct differences in band positions, time scales, and extinction coefficients upon excitation at 500 nm and 550 nm. It was reported that two chromophore conformations appear with all-*trans* and 13-*cis* retinal of 70 and 30% abundance, respectively. Raman stretching vibrations of the C = C were determined at 1533 cm^−1^ for *Ca*ChR1 with all-*trans* retinal and at 1550 cm^−1^ for *Ca*ChR1 with 13-*cis* retinal (Muders et al., 2014). Thus, the differences in the photoreaction dynamics can be explained by ground-state heterogeneity of the *Ca*ChR1 retinalchromophore.

We determined the absorption maxima of *Ca*ChR1 with all-*trans* retinal, and 13-*cis* retinal to be at 540, and ~480 nm, respectively. This is in accordance with the reported linear correlation between the frequency of the retinal C = C stretching vibration ν (C = C) and the maximum of the visible absorption spectrum λ_*max*_ for equilibrated ground-state structures (Aton et al., 1977; Fodor et al., 1989).

Excitation at 500 nm triggers the photoreaction of *Ca*ChR1 with all-*trans* retinal, and of *Ca*ChR1 with 13-*cis* retinal. After subtraction of the *Ca*ChR1 with all-*trans* retinal dynamics we observed the *Ca*ChR1 with 13-*cis* retinal. We found an ultrafast photoreaction with a positive signal around 600 nm, which decays on a time scale of <300 fs. This is indicated by the decay of the negative and positive signals in Figure 2C, S7A at early delay times around 480 and 600 nm, respectively. A significant positive signal of the photoproduct Q with a maximum around 590 nm indicates absorption, decaying with a time constant τ_2_ = (1.8 ± 0.3) ps. The remaining positive absorption Q' with maximum around 570 nm decays with τ_3_ = (90 ± 25) ps. The bleaching signals around 480 nm are weak compared to the strong positive signals around 600 nm, indicating a smaller extinction coefficient for the bleaching signals. This explains the poor dynamics of the bleaching signal that might overlap with the high-energy part of the Q spectrum of similar strength. Hence, bleaching recovery of the Q population result in negligible changes of the bleaching signal. The dynamics of *Ca*ChR1 with 13-*cis* retinal show three time constants also visible in the spectral integrated transient in Figure S4A. Thus, we expect all time constants to be connected with changes of electronic state properties.

Since the bleaching signals are the same for excitation at 550 and 500 nm after 100 ps, we expect the photoreaction of *Ca*ChR1 with 13-*cis* retinal to be recovered to its parent state at this time. Thus, several photoreaction mechanism can be discussed:

A photoreaction without isomerization would promote the *Ca*ChR1 13-*cis* ground state (*cis*) to its electronic excited state cis^*^, followed by a fast relaxation in the electronic excited state, decays to *cis'* ground state with vibrational excited protein surrounding, and relaxed back to its parent state *cis*. The first time constant of 200 fs (see Figure S7C) would correspond to the relaxation process, the second time constant τ_2_ = (1.8 ± 0.3) ps to the cis^*^ *→ cis'* transition, and the 90 ps time constant to the recovery of the parent state. The only remaining question is why is the extinction coefficient so different in cis^*^ and *cis'* compared to *cis*. It seems to be more plausible that the difference of extinction coefficient is due to different chromophore structures induces by isomerization.

Possible photoreactions with two isomerizations start with the promotion of the *Ca*ChR1 13-*cis* ground state (*cis*) to its electronic excited state cis^*^, followed by a fast relaxation in the electronic excited state. The first isomerization can occur either in the electronic excited state (cis^*^ → trans^*^), or can be accompanied by the transition from the excited to the ground state cis^*^ → trans ^*^ or trans^*^ → cis'. If a ground state with *trans'* is formed, an isomerization to *cis* has to take place in the ground state. The first time constant of 200 fs can be connected with an excited state relaxation or a cis^*^ → trans^*^ isomerization. The second time constant of 1.8 ps can be due to transitions from cis^*^ → trans' or trans^*^ → cis'. The third time constant of 90 ps reflects the recovery to the parent *Ca*ChR1 13-*cis* ground state from *trans' → cis* or *cis' → cis*.

Since isomerization processes in the electronic ground state are seldom, we prefer the photoreaction with a cis^*^ → trans ^*^ isomerization in the electronic excited state on a time scale of <300 fs inducing a strong signal change (Q), followed by a back-isomerization (trans^*^ → cis') with 1.8 ps accompanied with the transition from the electronic excited to the ground state cis' (Q'), that recovers to its parent state by 90 ps. A small fraction can also decay to a *trans'* ground state (trans^*^ → *trans*').

This would explain the positive signal above 520 nm, upon excitation at 500 nm, with a significantly higher extinction coefficient than upon excitation at 550 nm for long delay times. We tentatively assign this positive signal to a photoproduct *Ca*ChR1 with all-*trans* retinal. For Anabaena Sensory Rhodopsin, it was reported that excitation of the ground state with 13-*cis* retinal, leads to formation of a first K-like photoproduct with all-*trans* retinal, decaying back to ground state with all-*trans* retinal (Anderson et al., 2004). The photoreaction of *Ca*ChR1 with 13-*cis* retinal is completed after some 100 picoseconds. Whether this photoreaction induces dynamics and structural changes of the protein with possible biological function remains unclear, and has to be clarified in other studies.

In summary, we determined the primary photoreaction of *Ca*ChR1 for the first time. Ground-state heterogeneity of two isomers of the chromophore, all-*trans* retinal and 13-*cis* retinal leads to deviating photoreaction upon changing the excitation wavelength. Shorter wavelengths result in an increase of *Ca*ChR1 with 13-*cis* retinal dynamics, while excitation at longer wavelength increase the photoreaction of *Ca*ChR1 with all-*trans* retinal. Our data for *Ca*ChR1 with all-*trans* retinal are best explained by an all-*trans* to 13-*cis* retinal isomerization and hot photoproduct P_1_ formation with a time constant of ~100 fs. The photoreaction of *Ca*ChR1 with all-*trans* retinal turns out to be faster than in *Cr*ChR2, and exhibit strong oscillatory signals as reported for bacteriorhodopsin. Our data demonstrate a heterogeneity of isomers in the ground state with different photodynamics, but only one reaction pathway seems to be relevant for biological function.

### Conflict of interest statement

The authors declare that the research was conducted in the absence of any commercial or financial relationships that could be construed as a potential conflict of interest.

## References

[B1] Anderson S. Srajer V. Moffat K. (2004). Structural heterogeneity of cryotrapped intermediates in the bacterial blue light photoreceptor, photoactive yellow protein. Photochem. Photobiol. 80, 7–14. 10.1562/2004-03-15-RA-115.1 15339224

[B2] Aton B. Callender R. H. Becher B. Ebrey T. G. (1977). Resonance raman studies of purple membrane. Biochemistry 16, 2995–2999. 10.1021/bi00632a029 880292

[B3] Bamann C. Kirsch T. Nagel G. Bamberg E. (2008). Spectral characteristics of the photocycle of channelrhodopsin-2 and its implication for channel function. J. Mol. Biol. 375, 686–694. 10.1016/j.jmb.2007.10.072 18037436

[B4] Ernst O. P. Sanchez Murcia P. A. Daldrop P. Tsunoda S. P. Kateriya S. Hegemann P. (2008). Photoactivation of channelrhodopsin. J. Biol. Chem. 283, 1637–1643. 10.1074/jbc.M708039200 17993465

[B5] Fenno L. Yizhar O. Deisseroth K. (2011). The development and application of optogenetics. Annu. Rev. Neurosci. 34, 389–412. 10.1146/annurev-neuro-061010-113817 21692661 PMC6699620

[B6] Fodor S. P. A. Gebhard R. Lugtenburg J. Bogomolni R. A. Mathies R. A. (1989). Structure of the retinal chromophore in sensory rhodopsin-I from resonance Raman-spectroscopy. J. Biol. Chem. 264, 18280–18283. 2808377

[B7] Gervasio F. L. Cardini G. Salvi P. R. Schettino V. (1998). Low-frequency vibrations of all-trans-retinal: far-infrared and Raman spectra and density functional calculations. J. Phys. Chem. A 102, 2131–2136. 10.1021/jp9724636

[B8] Harbison G. S. Smith S. O. Pardoen J. A. Winkel C. Lugtenburg J. Herzfeld J.. (1984). Dark-adapted bacteriorhodopsin contains 13-cis, 15-syn and all-trans, 15-anti retinal Schiff bases. Proc. Natl. Acad. Sci. U.S.A. 81, 1706–1709. 10.1073/pnas.81.6.1706 6584904 PMC344987

[B9] Heyne K. Herbst J. Dominguez-Herradon B. Alexiev U. Diller R. (2000). Reaction control in bacteriorhodopsin: impact of arg82 and asp85 on the fast retinal isomerization, studied in the second site revertant arg82ala/gly231cys and various purple and blue forms of bacteriorhodopsin. J. Phys. Chem. B 104, 6053–6058. 10.1021/jp992877u

[B10] Heyne K. Huse N. Dreyer J. Nibbering E. T. J. Elsaesser T. Mukamel S. (2004b). Coherent low-frequency motions of hydrogen bonded acetic acid dimers in the liquid phase. J. Chem. Phys. 121, 902–913. 10.1063/1.1762873 15260622

[B11] Heyne K. Nibbering E. T. J. Elsaesser T. Petkovic M. Kuhn O. (2004a). Cascaded energy redistribution upon O-H stretching excitation in an intramolecular hydrogen bond. J. Phys. Chem. A 108, 6083–6086. 10.1021/jp048653f

[B12] Hou S. Y. Govorunova E. G. Ntefidou M. Lane C. E. Spudich E. N. Sineshchekov O. A.. (2012). Diversity of chlamydomonas channelrhodopsins. Photochem. Photobiol. 88, 119–128. 10.1111/j.1751-1097.2011.01027.x 22044280 PMC3253254

[B14] Johnson P. J. M. Halpin A. Morizumi T. Brown L. S. Prokhorenko V. I. Ernst O. P.. (2014). The photocycle and ultrafast vibrational dynamics of bacteriorhodopsin in lipid nanodiscs. Phys. Chem. Chem. Phys. 16, 21310–21320. 10.1039/C4CP01826E 25178090

[B15] Kato H. E. Zhang F. Yizhar O. Ramakrishnan C. Nishizawa T. Hirata K. (2012). Crystal structure of the channelrhodopsin light-gated cation channel. Nature 482, 369–374. 10.1038/nature10870 22266941 PMC4160518

[B16] Kim P. W. Freer L. H. Rockwell N. C. Martin S. S. Lagarias J. C. Larsen D. S. (2012). Femtosecond photodynamics of the red/green cyanobacteriochrome NpR6012g4 from Nostoc punctiforme. 1. Forward dynamics. Biochemistry 51, 608–618. 10.1021/bi201507k 22148715

[B17] Kovalenko S. A. Dobryakov A. L. Ruthmann J. Ernsting N. P. (1999). Femtosecond spectroscopy of condensed phases with chirped supercontinuum probing. Phys. Rev. A 59, 2369–2384. 10.1103/PhysRevA.59.2369

[B43] Kozich V. Dreyer J. Ashihara S. Werncke W. Elsaesser T. (2006). Mode-selective O-H stretching relaxation in a hydrogen bond studied by ultrafast vibrational spectroscopy. J. Chem. Phys. 125, 074504-1–074504-9. 10.1063/1.2219111 16942348

[B18] Krause N. Engelhard C. Heberle J. Schlesinger R. Bittl R. (2013). Structural differences between the closed and open states of channelrhodopsin-2 as observed by EPR spectroscopy. FEBS Lett. 587, 3309–3313. 10.1016/j.febslet.2013.08.043 24036447

[B19] Lorenz-Fonfria V. A. Heberle J. (2014). Channelrhodopsin unchained: structure and mechanism of a light-gated cation channel. Biochim. Biophys. Acta 1837, 626–642. 10.1016/j.bbabio.2013.10.014 24212055

[B20] Lorenz-Fonfria V. A. Muders V. Schlesinger R. Heberle J. (2014). Changes in the hydrogen-bonding strength of internal water molecules and cysteine residues in the conductive state of channelrhodopsin-1. J. Chem. Phys. 141, 22D507. 10.1063/1.4895796 25494778

[B21] Lorenz-Fonfria V. A. Resler T. Krause N. Nack M. Gossing M. Fischer von Mollard G.. (2013). Transient protonation changes in channelrhodopsin-2 and their relevance to channel gating. Proc. Natl. Acad. Sci. U.S.A. 110, E1273–E1281. 10.1073/pnas.1219502110 23509282 PMC3619329

[B22] Mailliet J. Psakis G. Feilke K. Sineshchekov V. Essen L. O. Hughes J. (2011). Spectroscopy and a high-resolution crystal structure of Tyr263 mutants of cyanobacterial phytochrome Cph1. J. Mol. Biol. 413, 115–127. 10.1016/j.jmb.2011.08.023 21888915

[B23] Mowery P. C. Lozier R. H. Chae Q. Tseng Y. W. Taylor M. Stoeckenius W. (1979). Effect of acid pH on the absorption spectra and photoreactions of bacteriorhodopsin. Biochemistry 18, 4100–4107. 10.1021/bi00586a007 39590

[B24] Muders V. Kerruth S. Lorenz-Fonfria V. A. Bamann C. Heberle J. Schlesinger R. (2014). Resonance Raman and FTIR spectroscopic characterization of the closed and open states of channelrhodopsin-1. FEBS Lett. 588, 2301–2306. 10.1016/j.febslet.2014.05.019 24859039

[B25] Muller M. Bamann C. Bamberg E. Kuhlbrandt W. (2011). Projection structure of channelrhodopsin-2 at 6 angstrom resolution by electron crystallography. J. Mol. Biol. 414, 86–95. 10.1016/j.jmb.2011.09.049 22001017

[B26] Muller M. Bamann C. Bamberg E. Kuhlbrandt W. (2015). Light-induced helix movements in channelrhodopsin-2. J. Mol. Biol. 427, 341–349. 10.1016/j.jmb.2014.11.004 25451024

[B27] Nack M. Radu I. Bamann C. Bamberg E. Heberle J. (2009). The retinal structure of channelrhodopsin-2 assessed by resonance Raman spectroscopy. FEBS Lett. 583, 3676–3680. 10.1016/j.febslet.2009.10.052 19854176

[B28] Nagel G. Ollig D. Fuhrmann M. Kateriya S. Musti A. M. Bamberg E.. (2002). Channelrhodopsin-1: a light-gated proton channel in green algae. Science 296, 2395–2398. 10.1126/science.1072068 12089443

[B29] Nagel G. Szellas T. Huhn W. Kateriya S. Adeishvili N. Berthold P.. (2003). Channelrhodopsin-2, a directly light-gated cation-selective membrane channel. Proc. Natl. Acad. Sci. U.S.A. 100, 13940–13945. 10.1073/pnas.1936192100 14615590 PMC283525

[B30] Neumann-Verhoefen M. K. Neumann K. Bamann C. Radu I. Heberle J. Bamberg E.. (2013). Ultrafast infrared spectroscopy on channelrhodopsin-2 reveals efficient energy transfer from the retinal chromophore to the protein. J. Am. Chem. Soc. 135, 6968–6976. 10.1021/ja400554y 23537405

[B31] Ogren J. I. Mamaev S. Russano D. Li H. Spudich J. L. Rothschild K. J. (2014). Retinal chromophore structure and Schiff base interactions in red-shifted channelrhodopsin-1 from *Chlamydomonas augustae*. Biochemistry 53, 3961–3970. 10.1021/bi500445c 24869998 PMC4072394

[B13] Ogren J. I. Yi A. Mamaev S. Li H. Spudich J. L. Rothschild K. J. (2015a). Proton transfers in a channelrhodopsin-1 studied by Fourier transform infrared (FTIR) difference spectroscopy and site-directed mutagenesis. J. Biol. Chem. 290, 12719–12730. 10.1074/jbc.M114.634840 25802337 PMC4432289

[B32] Ogren J. I. Yi A. Mamaev S. Li H. Lugtenburg J. DeGrip W. J.. (2015b). Comparison of the structural changes occurring during the primary phototransition of two different channelrhodopsins from *Chlamydomonas algae*. Biochemistry 54, 377–388. 10.1021/bi501243y 25469620 PMC4303311

[B33] Polli D. Altoe P. Weingart O. Spillane K. M. Manzoni C. Brida D.. (2010). Conical intersection dynamics of the primary photoisomerization event in vision. Nature 467, 440–443. 10.1038/nature09346 20864998

[B34] Prokhorenko V. I. Nagy A. M. Waschuk S. A. Brown L. S. Birge R. R. Miller R. J. D. (2006). Coherent control of retinal isomerization in bacteriorhodopsin. Science 313, 1257–1261. 10.1126/science.1130747 16946063

[B35] Rey R. Moller K. B. Hynes J. T. (2004). Ultrafast vibrational population dynamics of water and related systems: a theoretical perspective. Chem. Rev. 104, 1915–1928. 10.1021/cr020675f 15080716

[B36] Ritter E. Piwowarski P. Hegemann P. Bartl F. J. (2013). Light-dark adaptation of channelrhodopsin C128T mutant. J. Biol. Chem. 288, 10451–10458. 10.1074/jbc.M112.446427 23439646 PMC3624427

[B37] Ritter E. Stehfest K. Berndt A. Hegemann P. Bartl F. J. (2008). Monitoring light-induced structural changes of channelrhodopsin-2 by UV-visible and fourier transform infrared spectroscopy. J. Biol. Chem. 283, 35033–35041. 10.1074/jbc.M806353200 18927082 PMC3259888

[B38] Sattig T. Rickert C. Bamberg E. Steinhoff H. J. Bamann C. (2013). Light-induced movement of the transmembrane HelixB in channelrhodopsin-2. Angew. Chem. Int. Ed. Engl. 52, 9705–9708. 10.1002/anie.201301698 23893661

[B39] Schapiro I. Ruhman S. (2014). Ultrafast photochemistry of anabaena sensory rhodopsin: experiment and theory. Biochim. Biophys. Acta 1837, 589–597. 10.1016/j.bbabio.2013.09.014 24099700

[B40] Shigeto S. Pang Y. Fang Y. Dlott D. D. (2008). Vibrational relaxation of normal and deuterated liquid nitromethane. J. Phys. Chem. B 112, 232–241. 10.1021/jp074082q 17685649

[B41] Sineshchekov O. A. Govorunova E. G. Wang J. Li H. Spudich J. L. (2013). Intramolecular proton transfer in channelrhodopsins. Biophys. J. 104, 807–817. 10.1016/j.bpj.2013.01.002 23442959 PMC3576534

[B42] Sineshchekov V. A. (2004). Phytochrome A: functional diversity and polymorphism. Photochem. Photobiol. Sci. 3, 596–607. 10.1039/b315430k 15170491

[B44] Verhoefen M. K. Bamann C. Blocher R. Forster U. Bamberg E. Wachtveitl J. (2010). The photocycle of channelrhodopsin-2: ultrafast reaction dynamics and subsequent reaction steps. Chemphyschem 11, 3113–3122. 10.1002/cphc.201000181 20730849

[B45] von Stetten D. Gunther M. Scheerer P. Murgida D. H. Mroginski M. A. Krauss N.. (2008). Chromophore heterogeneity and photoconversion in phytochrome crystals and solution studied by resonance Raman spectroscopy. Angew. Chem. Int. Ed. Engl. 47, 4753–4755. 10.1002/anie.200705716 18484576

