# Supplementary material for: The primary photoreaction of channelrhodopsin-1: wavelength dependent photoreactions induced by ground-state heterogeneity
Source: Front Mol Biosci. 2015 Jul 22;2:41. doi: 10.3389/fmolb.2015.00041 (PMC4510425; doi:10.3389/fmolb.2015.00041)
Supplement: Supplementary file 1 [file DataSheet1.PDF]

Supplementary Material

The primary photoreaction of channelrhodopsin-1: Wavelength dependent photoreactions induced by ground-state heterogeneity

Till Stensitzki, Vera Muders, Ramona Schlesinger, Joachim Heberle, Karsten Heyne*

Freie Universität Berlin, Institut für Experimentalphysik, Arnimallee 14, 14195 Berlin, Germany

*** Correspondence:** Karsten Heyne, Freie Universität Berlin, Institut für Experimentalphysik, Arnimallee 14, 14195 Berlin, Germany.
Karsten.heyne@fu-berlin.de

Keywords: *Ca*ChR1, retinal, isomerization, femtosecond pump-probe spectroscopy, reaction model, ground state heterogeneity

1. Supplementary Data

Examination of the photoreaction quantum yield is difficult and can only be roughly estimated by our data. Upon excitation at 550 nm the DAS at 100 fs indicates no change of the ground-state bleaching signal, but a positive signal contribution of ~ 1.5 mOD at 510 nm (Figure 4). At the same time the absorption difference spectrum in Figure 5A displays a negative signal of ~ 9.5 mOD. This results in a bleaching signal of 11 mOD at 510 nm. For 100 ps the remaining bleaching signal is 2.8 mOD. From this ratio a maximal bleaching recovery, and a minimal quantum yield of ~ 0.25 can be estimated. This analysis uses directly the negative signal of the absorption difference at 100 ps delay time. Since the broad positive P_1 absorption band superimposes the bleaching band, the original contribution of the bleaching band should be higher. Assuming a P_1 absorption profile with a smooth shape connecting the positive contributions at 560 nm and 430 nm, we had to scale the bleaching contribution by 2.8 (Figure S1). This results in 2.8 fold contribution of the bleaching signal at 100 ps, and an increase of the quantum yield to ~ 70%. The same rough estimation leads to a quantum yield higher than ~ 25 % and smaller than ~ 40% for excitation at 500 nm. This can be explained by the additional 13-*cis* retinal reaction pathway that does not contribute to the forward reaction.

Figure S1: Calculated thermally relaxed photoproduct spectrum (blue line): The absorption difference at 100 ps (black line) is displayed together with an absorption spectrum of *Ca*ChR1. We scaled the absorption spectrum by 2.8, and added it to the absorption difference at 100 ps. The result was the photoproduct spectrum of P_1 (blue line). Smaller scaling factors lead to a double peak structure of the photoproduct with peaks at 560 nm and 440 nm.

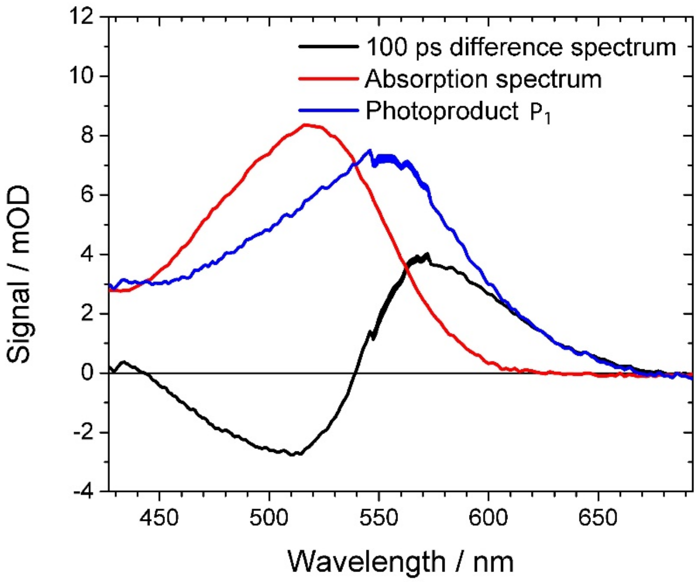

Figure S2: Contour plots of the photoreaction dynamics of *Ca*ChR1 upon excitation at 550 nm (A), 500 nm (B), and the difference of both datasets (C). Blue colors indicate negative signals, red colors positive signals. The contour plots display the absorbance difference in mOD upon excitation as a function of delay time and wavelength. The difference contour plot (C) is calculated by the direct difference of (B)-(A).

.

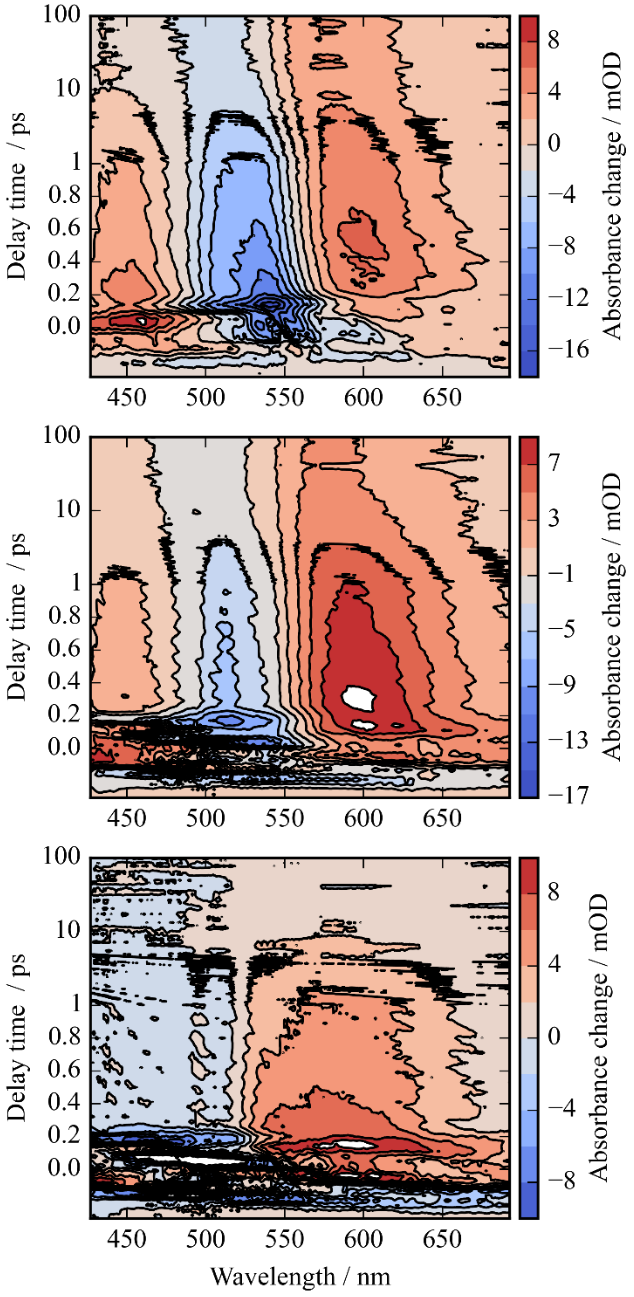

Figure S3: FFT amplitudes of the residues as a function of wavelength and wavenumber for a time window of 3 ps. Contributions around 7cm^{-1}, 100 cm^{-1}, 150 cm^{-1}, 200 cm^{-1}, and 225 cm^{-1} are clearly visible.

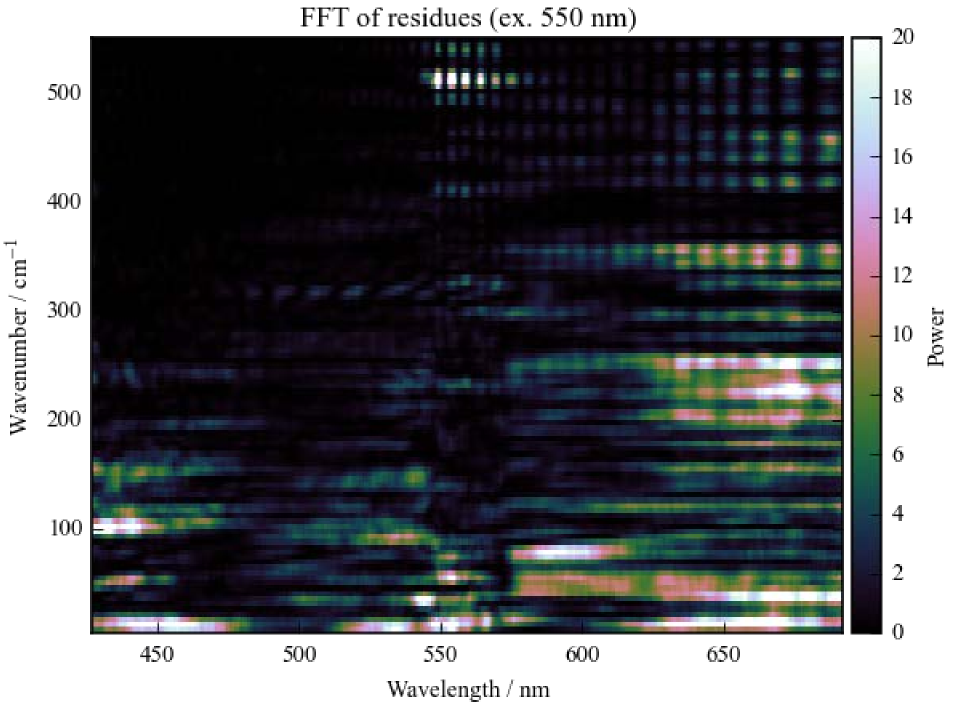

Figure S4: Spectral integrated signals upon excitation at 500 nm (A),(B), and at 550 nm (C). For all integrations the spectral axis was transferred to wavenumbers and the different spectral resolutions were taken into account. (A) T(1)ransient of the complete spectral integrated signal (blue line), and a simulation (orange line) with time constants of (170±70) fs, (1.3±0.5) ps, and 30 ps. The strong coherent oscillations around time zero are dominated by nonlinear signals of the CaF_2 windows. (B) All positive, and negative signals of the dataset were spectral integrated; the P/N transient (blue line) is the ratio of the integrated positive to integrated negative transient. P/N signals are sensitive to spectral shifts of bands with different extinction coefficient, while the integrated signal is not. The sub picosecond and picosecond time components are clearly visible, as well as oscillatory behavior with a period of 100 fs. (C) Integrated positive (black line), integrated negative (red line), and integrated P/N transient (green line) plotted as a function of delay time. The P/N transient exhibits a small signal increase up to 500 fs and stays nearly constant for longer delay times.

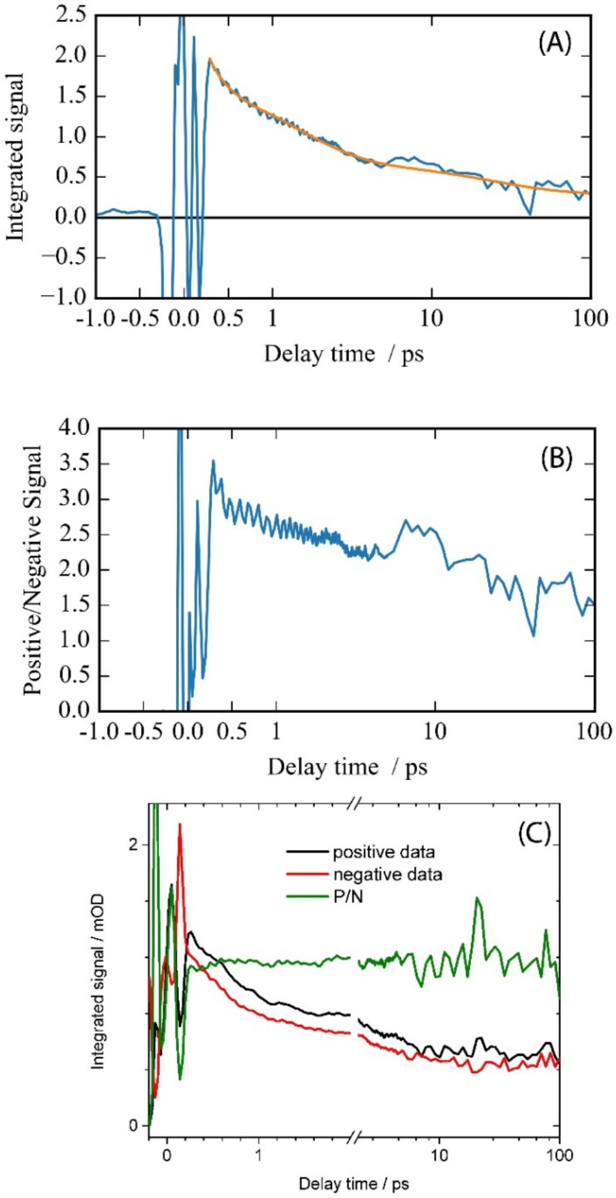

Figure S5: Absorption difference spectra at 100 ps upon excitation at 500 nm (blue line), 550 nm (orange line), and its difference scaled by a factor of 3 (green line). The shape of the bleaching signal is nearly identical from 430 nm to 510 nm, indicating not remaining bleaching signal from *Ca*ChR1 with 13-*cis* retinal. The difference shows signatures of a broad absorbing species with maximum around 610 nm.

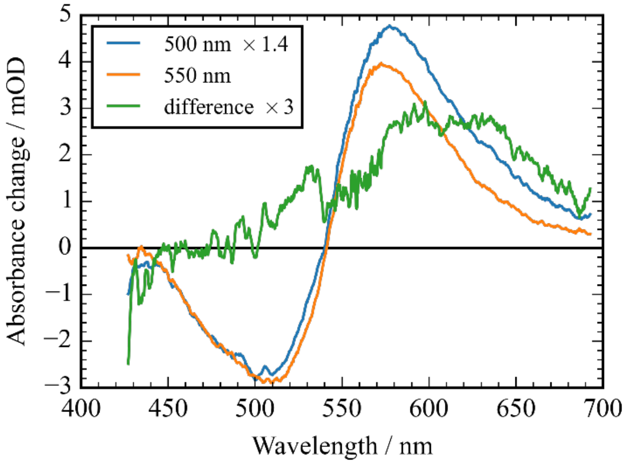

500 nm × 1.4
550 nm
difference × 3
Absorbance change / mOD
Wavelength / nm
5
4
3
2
1
0
−1
−2
−3
400
450
500
550
600
650
700

Figure S6: Nonlinear signal in the CaF_2 windows at 550 nm probe wavelength. The “glass signal” (blue dots) represents the nonlinear signal of the empty sample cell with CaF_2 windows. The signal can be well simulated (orange line) by a Gaussian ($\exp(-t^2/\sigma^2)$) and its first and second derivative as reported by Kovalenko et al.(1) This simulation results at an instrument response function (IRF) and cross-correlation of σ=90 fs. Note, using a standard Gaussian ($\exp(-t^2/[2\sigma^2])$) would result in an even shorter IRF. Hence, the time resolution, given by the IRF or cross-correlation is better than 100 fs. Therefore, small oscillatory features of the ~320 cm^{-1} vibration with an oscillation period of 104 fs of the windows are visible, only possible with an IRF below 100 fs.

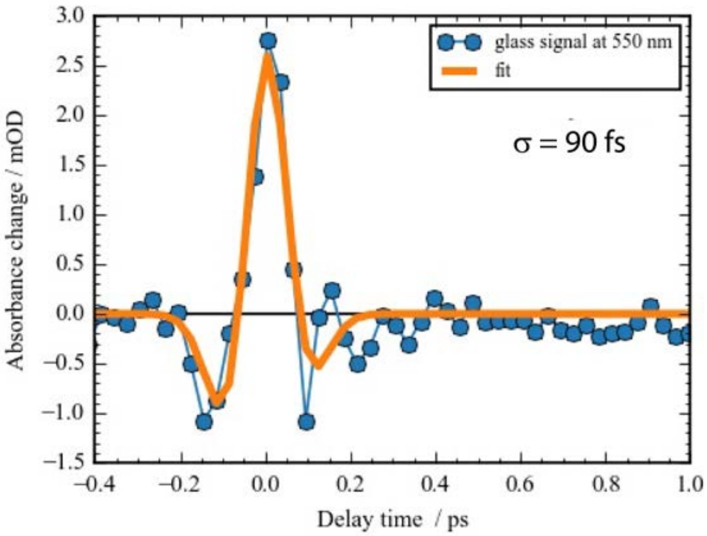

Figure S7: Dynamics of *Ca*ChR 1 with 13-*cis* retinal derived from the data by direct subtraction of the dataset excited at 500 nm from the dataset excited at 550 nm. Positive signals are new emerging bands, while negative signals show bleaching bands and stimulated emission. (A) Absorbance difference spectra for different delay times. For 100 ps delay time a positive signal remains around 650 nm, indicating the difference between 500 nm and 550 nm excitation at 100 ps delay time (Figure S5, green signal). (B) Transients at selected wavelengths on a logarithmic time scale. Three time constants are well visible. Solid lines represent the fits with three time constants of τ_1=200 fs, τ_2=1.8 ps, and τ_3=90 ps. (C) Decay associated spectra. The positive bands are stronger than the negative bands indicating higher extinction coefficients for the new emerging bands compared to the bleaching band.

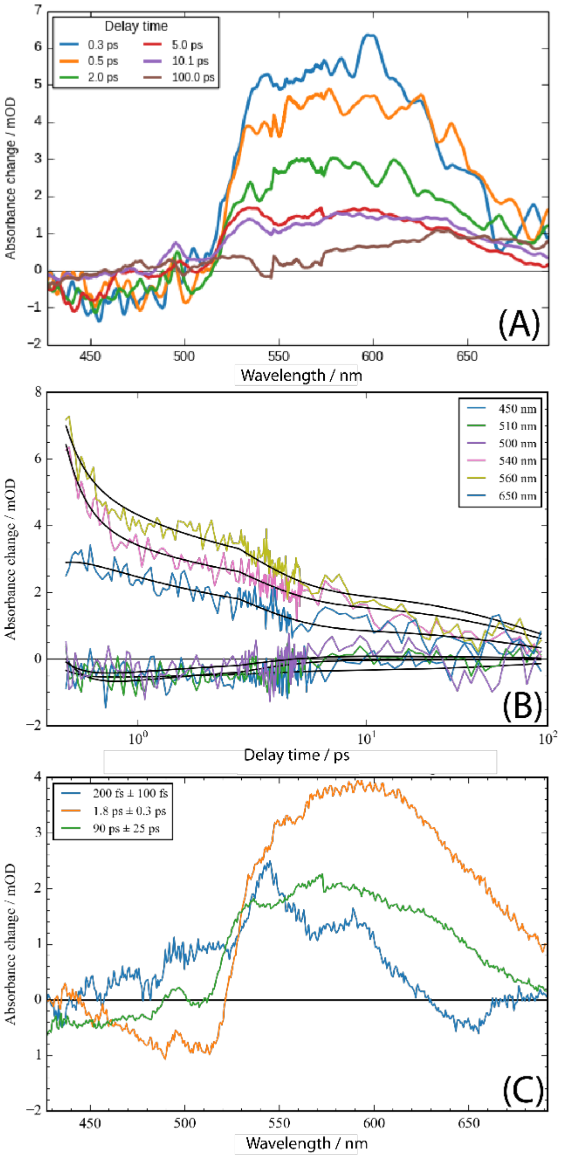

1. Kovalenko SA, Dobryakov AL, Ruthmann J, Ernsting NP. Femtosecond spectroscopy of condensed phases with chirped supercontinuum probing. *Phys Rev A* (1999) **59**:2369-84.
